# *Nocardia neocaledoniensis* as Rare Cause of Spondylodiscitis

**DOI:** 10.3201/eid2902.221389

**Published:** 2023-02

**Authors:** Emeline Choquet, Veronica Rodriguez-Nava, François Peltier, Rodrigue Wankap-Mogo, Emmanuelle Bergeron, Cédric Joseph, Nadine Lemaitre

**Affiliations:** Centre Hospitalier Universitaire, Amiens, France (E. Choquet, F. Peltier, R. Wankap-Mogo, C. Joseph, N. Lemaitre);; Université Lyon 1, Lyon, France (V. Rodriguez-Nava, E. Bergeron).

**Keywords:** Nocardia neocaledoniensis, spondylodiscitis, DNA sequencing, 16S rRNA gene, bacteria, France

## Abstract

*Nocardia neocaledoniensis* is a rare species of *Nocardia* bacteria, identified in 2004 in hypermagnesian ultramafic soil of New Caledonia. Culture of this opportunistic pathogen from spinal biopsy samples confirmed *N. neocaledoniensis* spondylodiscitis in an immunocompromised man. Isolation of this unusual species from spinal biopsy samples illustrates its underappreciated ability to cause invasive infection.

*Nocardia* are aerobic bacteria, order *Corynebacteriales* (*1*), that are ubiquitously found in soil and water. *Nocardia* are typically opportunistic pathogens in immunocompromised patients (*2*). *Nocardia neocaledoniensis* is responsible for skin and soft tissue infections (*3**,**4*)*;* its involvement in invasive infections has been 1 brain abscess and 1 case of bacteremia (*5**,**6*). We report a case of spondylodiscitis caused by *N. neocaledoniensis*.

In December 2021, in Amiens, France, a 68-year-old man with diabetes sought care from a rheumatologist after a 2-month history of persistent low-back pain radiating to his right hip and markedly reducing his ability to walk. He had a medical history of rectal cancer diagnosed 1 year earlier, for which liver metastasis was treated by surgery and 13 cycles of chemotherapy during March–September 2021.

The patient was apyretic, but his general status was deteriorated (asthenia, weight loss, and anorexia). Examination revealed marked tenderness over his mid-lumbar spine at L2–L3 and stiffness of the lumbar spine. Clinically relevant results of blood analysis showed elevated erythrocyte sedimentation rate (94 mm/h) and C-reactive protein (16 mg/L). A computed tomography (CT) scan showed erosion and end-plate deterioration of L2–L3, and a magnetic resonance imaging scan confirmed the diagnosis (Figure). Results of blood cultures were negative, but results of culture of 2 spinal biopsy samples collected during CT were positive in brain–heart infusion broth after 72 h of incubation at 37°C. Direct gram-stained smears showed unexpected branched gram-positive rods. After 48 h of subculture on a blood agar plate under aerobic conditions, the culture showed white opaque and dry *Nocardia*-like colonies. Matrix-assisted laser desorption/ionization time-of-flight (MALDI-TOF) mass spectrometry (Bruker, https://www.bruker.com) identified *N. asteroides*, with a score of 1.87*.* Sanger sequencing of the 16S rRNA (1,120 bp) gene from the isolate (GenBank accession no. OP028079) did not adequately discriminate between *N. neocaledoniensis* and *N. asteroides*. Therefore, we determined nucleotide sequences of the *secA1* (468 bp) and *sodA* (386 bp) genes and compared them with those from reference strains, as previously described (*7*,*8*). The isolate from the patient showed 99.1% similarity to *secA* and 99.5% to *sodA* sequences of the *N*. *neocaledoniensis* DSM 44717^T^ strain compared with <97.6% similarity to the *N. asteroides* ATCC 19247^T^ strain (Appendix Figure).

**Figure:**
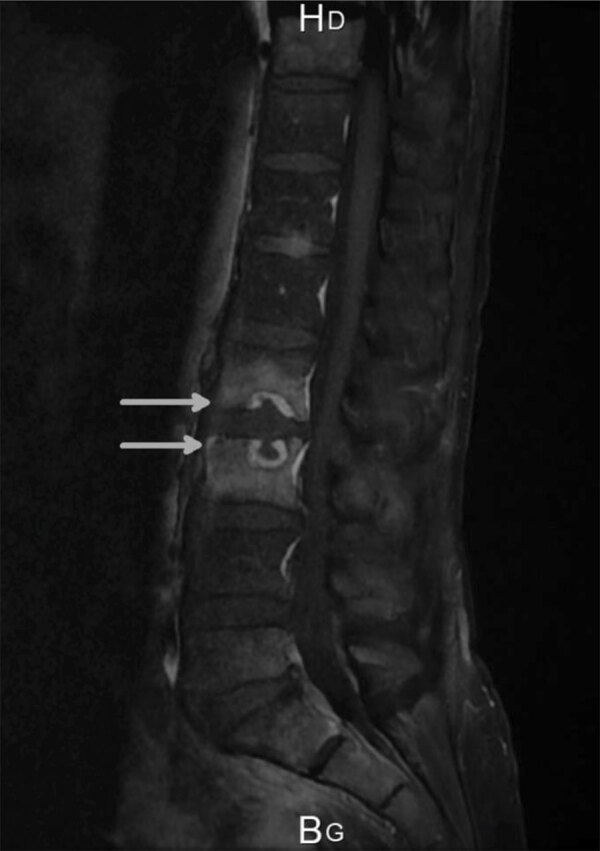
Sagittal gadolinium magnetic resonance image of spine of a patient in France with spondylodiscitis caused by *Nocardia neocaledoniensis*. Image shows erosion of the cortical end plate of L2 and L3 (arrows).

We performed susceptibility testing according to Clinical and Laboratories Standards Institute 2018 recommendations by using Sensititer RAPMYCO AST Plate (ThermoFisher Scientific, https://www.thermofisher.com). Based on the results of susceptibility testing (Table), treatment was intravenous ceftriaxone (2 g 2×/d) plus oral trimethoprim/sulfamethoxazole (160/800 mg 6×/d) for 1 week, followed by oral tedizolid (200 mg 1×/d). After 3 weeks of antimicrobial chemotherapy, the low-back pain completely regressed and the patient was mobile; after 6 months (antimicrobial therapy completion), the radiologic findings had slowly improved, and the patient was considered cured. 

**Table:** Antimicrobial susceptibility of *Nocardia neocalendoniensis* interpreted according to Clinical and Laboratory Standards Institute breakpoints (*7*)*

Antimicrobial	MIC, mg/L	Susceptibility
Amoxicillin/clavulanic acid	>64/32	R
Ceftriaxone	4	S
Imipenem	4	S
Amikacin	<1	S
Tobramycin	<1	S
Linezolid	2	S
Trimethoprim/sulfamethoxazole	0.5/9.5	S
Minocycline	4	I
Doxycycline	8	R
Ciprofloxacin	>4	R
Moxifloxacin	2	I
Clarithromycin	16	R

*I, intermediate; R, resistant; S, susceptible.

Immunosuppressive therapy, which decreases cellular immunity, is often associated with nocardiosis (*2*). The cancer patient we report had received immunosuppressants for 7 months. Common clinical manifestations are pulmonary infection caused by inhalation of *Nocardia* in aerosols and cutaneous infection after penetrating injuries (*2*). Both primary infection sites could be considered for this patient, who is a gardener. Indeed, the systematic thoracic CT scan performed in September 2021 showed ground-glass opacities with 2 excavations in the left upper lobe, which might be interpreted as manifestations of pulmonary nocardiosis, and the patient reported having had hand injuries 4–5 months before the diagnosis. Because the patient was asymptomatic, no microbiological specimens were collected before December 2021; he was twice tested for SARS-CoV-2 by PCR, in October and December, and results were negative. The cause of the pneumonia could not be determined, and the primary infection site of nocardiosis remains unknown for this patient.

Molecular methods based on housekeeping genes (16S rRNA, *hsp65*, *rpoB*, *gyrB*, *sodA*, and *secA1*) DNA sequences have characterized ≈100 species of *Nocardia* (*7**, **8*). For identifying *Nocardia*, MALDI-TOF mass spectrometry is more rapid and cost-effective than molecular methods. Several studies reported correctly identifying *Nocardia* to the species level (≈95%) by using commercial databases with common species (*9**,**10*). We used MALDI-TOF mass spectrometry with the Bruker reference library version 9.0, updated with 8,326 main spectra profiles. This database includes 52 *Nocardia* species but not *N. neocaledoniensis*, unlike the Vitek MD database (bioMérieux, http://www.biomerieux.com). Because *N. neocaledoniensis* is closely related to *N. asteroides* (*7*), the isolate was misidentified (with a low score of 1.87) as *N. asteroides* with the Bruker library. Although sequencing of 16S rRNA is usually adequate for identifying *Nocardia* isolates, in this case, the combined sequence analysis of *secA* and *sodA* genes was required to accurately discriminate between the closely related *N. neocaledoniensis* and *N. asteroides*.

This report of spondylodiscitis caused by *N. neocaledoniensis* provides evidence that *N. neocaledoniensis* is an opportunistic pathogen involved in invasive infection that may be underdiagnosed. Indeed, few commercial MALDI-TOF mass spectrometry databases correctly identify this uncommon species; thus, enlarging of most databases is necessary. Until then, molecular biology-based methods should still be considered the best standard for accurate identification.

Appendix Supplementary methods used for study of *Nocardia neocaledoniensis* as rare cause of spondylodiscitis.
