# Supplementary material for: Nocardia neocaledoniensis as Rare Cause of Spondylodiscitis
Source: Emerg Infect Dis. 2023 Feb;29(2):444–6. doi: 10.3201/eid2902.221389 (PMC9881783; doi:10.3201/eid2902.221389)
Supplement: Appendix — Supplementary methods used for study of Nocardia neocaledoniensis as rare cause of spondylodiscitis. [file 22-1389-Techapp-s1.pdf]

Article DOI: https://doi.org/10.3201/eid2902.221389

# *Nocardia neocaledoniensis* as Rare Cause of Spondylodiscitis

## Appendix

**Appendix Table.** Primers used in this study for the DNA sequencing

| Gene and primers | Sequences (5′ →3′) | Reference |
|---|---|---|
| 16S rRNA | | (*1*,*2*) |
| VIV8F | AGAGTTTGATCMTGGCTCAG | |
| V1V8R | GGGCGGWGTGTACAAGGC | |
| V3V4F | CGGCCCAGACTCCTACGGGAGGCAGCA | |
| V3-V4R | GCGTGGACTACCAGGGTATCTAATCC | |
| Noc1F | GCTTAACACATGCAAGTCG | |
| Noc2R | GAATTCCAGTCTCCCCTG | |
| *sodA* | | (*3*) |
| SODF | CACCAYWSCAAGCACCA | |
| SODR | CCTTGACGTTCTGGTACTG | |
| *secA1* | | (*4*) |
| M13F | GTAAAACGACGACCAGGACAGYGAGTGGATGGGYCGSGTGCACCG | |
| M13R | CAGGAAACAGCTATGCGGCGGACGATGTAGTCCTTGTC | |

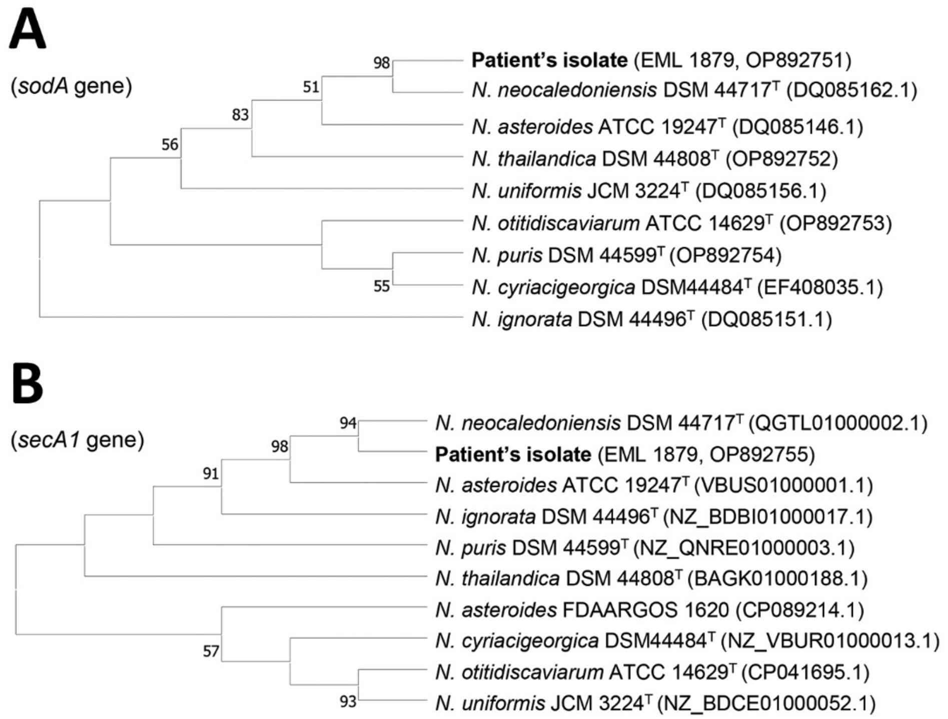


**Appendix Figure**. Alignment of 386 bp of the *sodA* (A) and 468 bp of the *secA1* genes (B) of the patient's isolate (strain EML 1879) with the *sodA* and *secA1* genes sequences of a set of reference strains of *Nocardia* species according to the Sánchez-Herrera's instructions (*5*). The evolutionary history

was inferred using the Neighbor-Joining method, Kimura's two-parameter model and bootstrap of 1000. Branches corresponding to partitions reproduced in less than 50% bootstrap replicates were collapsed.

## References


1. Sune D , Rydberg H , Augustinsson AN,  Serrander L, Jungeström MB. Optimization of 16S rRNA gene analysis for use in the diagnostic clinical microbiology service. J Microbiol Methods. 2020;170:105854.

2. Rodríguez-Nava V, Couble A, Devulder G, Flandrois JP, Boiron P, Laurent F. Use of PCR-restriction enzyme pattern analysis and sequencing database for *hsp65* gene-based identification of *Nocardia* species. J Clin Microbiol. 2006;44:536–46. PubMed https://doi.org/10.1128/JCM.44.2.536-546.2006

3. Sánchez-Herrera K, Sandoval H, Mouniee D, Ramírez-Durán N, Bergeron E, Boiron P, et al. Molecular identification of *Nocardia* species using the *sod*A gene: identificación molecular de especies de *Nocardia* utilizando el gen *sodA.* New Microbes New Infect. 2017;19:96–116. PubMed https://doi.org/10.1016/j.nmni.2017.03.008

4. Conville PS, Zelazny AM, Witebsky FG. Analysis of *secA1* gene sequences for identification of *Nocardia* species. J Clin Microbiol. 2006;44:2760–6. PubMed https://doi.org/10.1128/JCM.00155-06

5. Sánchez-Herrera K, Sandoval H, Mouniee D, Ramírez-Durán N, Bergeron E, Boiron P, et al. Molecular identification of *Nocardia* species using the *sod*A gene: Identificación molecular de especies de *Nocardia* utilizando el gen *sodA.* New Microbes New Infect. 2017;19:96–116. PubMed https://doi.org/10.1016/j.nmni.2017.03.008
